# The prognosis of gliomas with different molecular subtypes in the era of intensity-modulated radiation therapy (IMRT)

**DOI:** 10.18632/aging.204942

**Published:** 2023-08-08

**Authors:** Qiulu Zhong, Danjing Luo, Da Chen, Xiangde Li, Qinghua Du, Qianfu Liang, Jian Li, Xiaodong Zhu

**Affiliations:** 1 Department of Radiation Oncology, Guangxi Medical University Cancer Hospital, Nanning, Guangxi 530000, P.R. China; 2 Department of Radiation Oncology, The Second Affiliated Hospital of Guangxi Medical University, Nanning, Guangxi 530000, P.R. China; 3 Department of Oncology, Wuming Hospital of Guangxi Medical University, Nanning, Guangxi 530000, P.R. China

**Keywords:** gliomas, molecular subtypes, intensity-modulated radiation therapy (IMRT), isocitrate dehydrogenase (IDH)

## Abstract

Purpose: This study aimed to evaluate the prognosis of glioma patients with different molecular subtypes of who treated with intensity-modulated radiation therapy (IMRT).

Methods: We collected 45 glioma patients treated in our hospital between January 2017 and December 2020. All enrolled patients received postoperative IMRT and were divided into two groups based on the Isocitrate dehydrogenase (IDH status). Overall survival (OS) and progression-free survival (PFS) were estimated retrospectively.

Results: The median follow-up was 22 months (range 2–108.5 months). The 1-year OS of IDH-mut group and ΙDH-wild group was similar (77.3% vs. 81.5%, *p* = 0.16). While the 1-year PFS of IDH-mut group was significantly higher than that in ΙDH-wild group (90.4% vs. 39.8%, *p* = 0.0051). Subgroup analysis revealed that the 1-year PFS of IDH-mut/1p/19q codeletion group and IDH-mut/1p/19q noncodeletion group was significantly higher than in IDH-wild type patients. For patients with IDH-mut/MGMT-methylation, the outcome was no significant difference in OS, but PFS was longer than other subtypes.

Conclusion: This retrospective study showed that 1-year PFS of patients with IDH mutated was better than IDH-wild type patients. In subgroups analysis, the outcomes were shown that patients with IDH-mut/ 1p/19q codeletion and patients with IDH-mut/1p/19q noncodeletion had longer 1-year PFS than IDH-wild type patients, but the OS was similar between the subgroups. Patients with IDH-mut/MGMT-methylation had the best prognosis in the whole subgroups. However, these results still need further confirmation of large sample size, prospectively, randomized controlled trails.

## INTRODUCTION

Glioma is one of the most common malignant central nervous system tumor. According to central brain tumor registry of the United States (CBTRUS), glioma account for 27% of all CNS and approximately 80% of malignant tumors [1]. The treatment of glioma combines surgery, radiotherapy, chemotherapy, targeted therapy and other treatments according to the latest NCCN guidelines [2]. Due to the aggressive behavior, gliomas have high recurrence rate and mortality rate, so postoperative radiotherapy becomes particularly important, especially for high-grade glioma patients, postoperative radiotherapy can reduce the recurrence rate and prolong survival time. However, even if these patients received standard postoperative care, the 1-year overall survival (OS) still only 14.4 months [3]. Therefore, how to improve OS and reduce the local recurrence rate has become the research direction of many neuro-oncologists. Since on the 2016 World Health Organization (WHO) fourth revised edition proposed adding molecular typing as an important basis element for glioma diagnosis [4], the diagnosis, classification, prognosis and treatments of gliomas made great progress. The 5th edition of World Health Organization (WHO) classification of Central Nervous System Tumors released in 2021 [5] integrated the histological characteristics and molecular classification of glioma, and proposed a new tumor classification standard, which provides an important basis for the diagnosis, treatment and the classification of glioma. The new classification is no longer based on the cross solid tumors, but based on tumor phenotype, molecular subtype and biological similarity, which is more individualized than the previous classification based on the tumor gross type. Radiotherapy, as one of the most important postoperative treatment methods for high- grade gliomas, can bring significant survival benefits to patients. Previous studies [6, 7] have shown that the survival time of high-grade gliomas is closely related to the start time of radiotherapy, and early postoperative treatment can effectively prolong the survival time of patients with glioma. Early in 1996, Karim AB et al. [8] compared the efficacy of low-dose and high dose radiotherapy for low-grade gliomas, the results showed that there was no significantly difference between the two groups. In 2002, Shaw E et al. [9] carried out a randomized phase III study about the efficacy of low-dose (50.4 Gy/28f) compared with high-dose (64.8 Gy/36f) radiotherapy for low-grade gliomas. The outcomes revealed that patients who received higher doses of radiation had lower overall survival than those who received lower doses. Moreover, the incidence of radiation brain necrosis in high-dose radiotherapy group was also higher than low-dose radiotherapy group. The standard treatment for glioblastoma is STUPP regimen [10]. Despite patients treated with the standard method of STUPP, the median overall survival still only 14 months [11]. Walker MD et al. [12] found the dose-effect relationship in the radiotherapy of malignant gliomas in 1979. Bleehen NM et al. [13] also compared the efficacy of different radiation doses of 45 Gy and 60 Gy on the grades 3 and 4 astrocytomas. The results also confirmed that patients treated with 60 Gy had better prognosis. Is boosting the dose of local radiotherapy beneficial to patients with high-grade gliomas? A study by RTOG /EORTG showed boosting radiation dose to 70 Gy also didn’t benefit patients either [14]. Similarly, a randomized phase III study 93–05 by RTOG also revealed that boosting dose (15 to 24 Gy × 1f) on the basis of 60 Gy still didn’t bring benefit to patients [15]. Piroth MD et al. [16] integrated boost IMRT with FET-PET to delineate the radiotherapy target volumes in glioblastoma patients, and boosted the radiation dose to 72 Gy, but the outcomes still didn’t improve patients’ survival rate. Isocitrate dehydrogenase (IDH), is a key rate-limiting enzyme in tricarboxylic acid cycle. Some studies had confirmed that high-grade glioma patients with IDH mutation had better prognosis [17–20], but the prognostic value of IDH for low-grade diffuse glioma is still unclear [21]. However, most of the previous researches on the prognosis of new molecular subtypes of gliomas are based on conventional radiation or 3-dimensional conformal radiation (3D-CRT), what about intensity modulated radiation therapy (IMRT)? what’s the prognosis of patients with different molecular types in the era of IMRT? Do advanced radiation techniques benefit patients with different molecular classifications of glioma, or do patients with different molecular subtypes of glioma have inherently poor outcomes? There were rare studies about prognosis of patients with different molecular subtypes gliomas treated with IMRT. Therefore, our study retrospectively collected glioma patients based on the molecular subtypes who treated in our centre only received IMRT after surgery, and explored the prognosis of different molecular subtypes of gliomas.

## MATERIALS AND METHODS

### Patient population

We retrospectively collected glioma patients diagnose in the Second Affiliated Hospital of Guangxi Medical University from January 2017 to December 2020. The inclusion criteria were as follows: (1) Age between 16 and 70 years old; (2) histopathology confirmed astrocytoma, oligodendroglioma, glioblastoma, anaplastic astrocytoma, anaplastic oligodendroglioma; (3) Assessable IDH1,IDH2,MGMT,1p/19q status; (4) CT or MRI before surgery; (5) Didn’t receive radiotherapy, chemotherapy or other treatments before; (6) Double cancer were excluded; (7) ECOG score ≤2; (8) All the inclusion patients received post-radiotherapy; The exclusion criteria were as follows: (1) Patients who received tumor treated as before; (2) More than two types malignant tumors; (3) Incomplete follow-up data.

**Figure 1:**
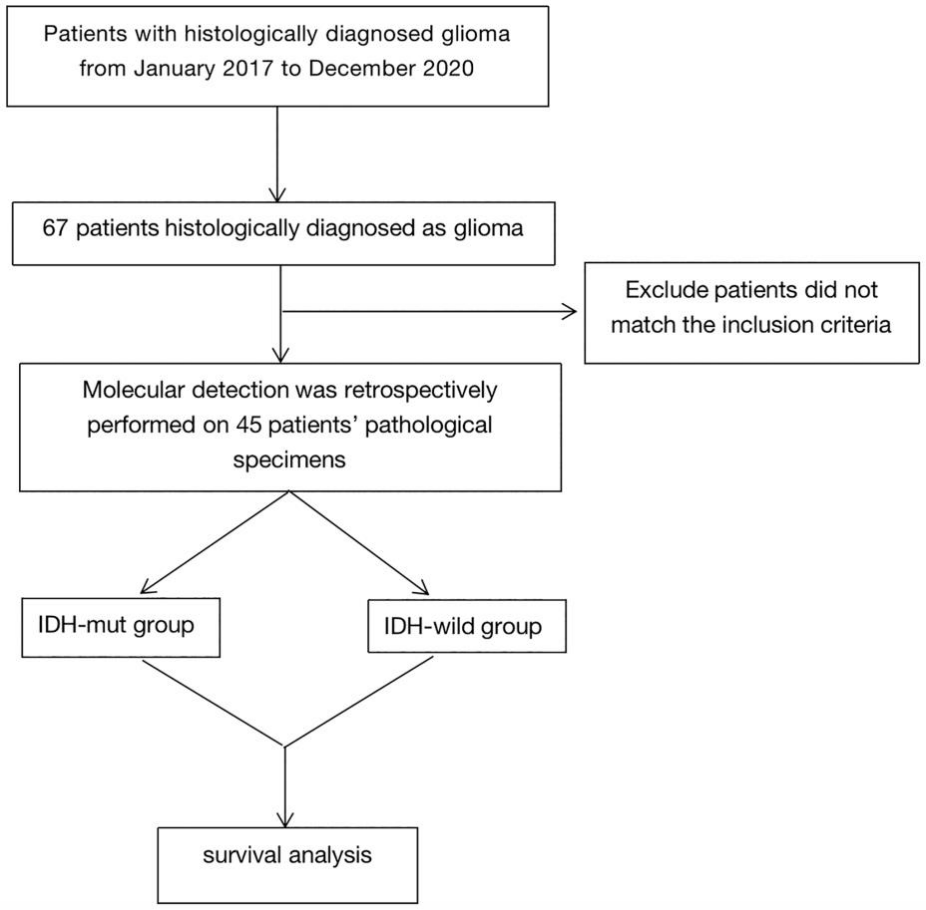
Flow chart.

Clinical data on patients’ age, gender, pathological type, WHO grade, preoperative tumor size, surgical resection method, radiotherapy dose, recurrence site from the medical history system of The Second Affiliated Hospital of Guangxi Medical University. All the patients received radiotherapy 4–8 weeks after surgery. According to the status of IDH, we divided enrolled patients into two groups: IDH mutate group (IDH-mut group) and IDH wild group (IDH-wild group), regardless their histopathology status. Patient characteristics were summarized in Table 1, the flow chart was shown in Figure 1.

**Table 1:** Characteristics of glioma patients stratified by IDH status.

**Characteristic**	**IDH-mut (%)**	**IDH-wild (%)**	***P*-value**
**No. of patients**	22 (48.9)	23 (51.1)	
**Median age**	42 (30 to 70)	47 (25 to 57)	0.939
**Sex**
Male	14 (63.6)	16 (69.6)	0.673
Female	8 (36.4)	7 (30.4)	
**WHO stage**
II	7 (31.8)	4 (17.4)	0.279
III	8 (36.4)	6 (26.1)	
IV	7 (31.8)	13 (56.5)	
**ECOG**
0	4 (18.2)	0 (0)	0.049
1	18 (81.8)	23 (100)	
**Tumor site**
Temporal lobe	9 (40.9)	9 (39.1)	0.564
Frontal lobe	7 (31.8)	9 (39.1)	
Cerebellum	1 (4.5)	0 (0)	
Parietal lobe	5 (22.7)	3 (13.0)	
Occipital lobe	0 (0)	2 (8.7)	
**Pathology**
Astrocytoma	7 (31.8)	4 (17.4)	0.209
Anaplastic oligodendroglioma	2 (9.1)	0 (0)	
Anaplastic astrocytomas	6 (27.3)	6 (26.1)	
Glioblastoma	7 (31.8)	13 (56.5)	
**Tumor size**
>6	9 (40.9)	10 (43.5)	0.551
<6	13 (59.1)	13 (56.5)	
**Surgical resection**
Total resection	9 (40.9)	4 (17.4)	0.175
Subtotal resection	12 (54.6)	18 (78.3)	
Biopsy	1 (4.5)	1 (4.3)	
**MGMT status**
MGMT-methylation	14 (63.6)	8 (34.8)	0.053
MGMT-unmethylation	8 (36.4)	15 (65.2)	
**Concurrent-Chemotherapy**
Yes	17 (77.3)	23 (100)	0.022
No	5 (22.7)	0 (0)	
**Adjuvant-Chemotherapy**
Yes	8 (36.4)	11 (478)	0.550
No	14 (63.6)	12 (52.2)	

### Molecular analysis

DNA was extracted from tumor tissues by standard methods, and all the samples were examined by experienced pathologists to ensure every sample had 80% or more tumor cell content. The techniques to determine MGMT promoter methylation by one-stage methylation-specific PCR [22] (methylation-specific primers generated a band on agarose gel in the lane containing PCR products was scored as positive). Frozen samples were used for IDH1 or IDH2 analysis. The genomic region spanning wild-type R132 of IDH1 was analyzed by direct sequencing using the following primers:5′TGTGTTGAGA TGGACGCCTATTTG and 3′TGCCACCAACGA CCAAGTC as the manufacture’s protocol. The genomic region spanning wild-type R172 of IDH2 was analyzed by direct sequencing using the following primers: IDH2f 5-GCCCGGTCTGCCACAAAGTC and IDH2r 5-TTGGCAGACTCCAGAGCCCA as the manufacture’s protocol.1p/19q codeletion was excluded by fluorescence *in situ* hybridization (FISH).

### Radiotherapy

All the enrolled patients were received 6 MV-X ray liner accelerator of intensity modulated radiation therapy (IMRT). Patients were immobilized with an individual head-neck-shoulder thermoplastic mask in the supine position to ensure reproducibility of patient positioning during planning CT and the following radiation. The slice thickness was 3 mm of the CT scan.

### Target volumes

The target volumes were administered according to the protocol of the EORTC and RTOG. Target delineation as follows:

#### Protocol of EORTC

The gross tumor volume (GTV) included the contrast-enhancing regions which present on T1-weighted preoperative MRI scans and all the surgical areas. The clinical target volume (CTV) was determined by the GTV with a margin up to 2 cm. The planning target volume (PTV) was created by extending a 3 to 5 mm margin of the corresponding CTV.

#### Protocol of RTOG

The gross tumor volume-1 (GTV-1) as the EORTC described before, but GTV-1 included the perifocal edema regions which was visible on T2 or FLAIR sequence. The clinical target-1 (CTV-1) was based on the GTV with a margin of up to 2 cm (extending 2.5 cm margin if it didn’t have perifocal edema). The planning target volume-1 (PTV-1) was the CTV1 extended 3 to 5 mm margin. The gross tumor volume-2 (GTV-2) included the surgical regions and all the contrast-enhancing regions detected on T1-weighted MRI. CTV-2 defined as the GTV-2 extended 2 cm margin. The planning target volume-2 (PTV-2) was 3 to 5 mm CTV2 margin expansion.

The prescription dose were as follows: the patients who diagnosed with low-grade gliomas treated with 54 Gy, but if patients had visible residual tumor on MRI, the dose boosted to 60 Gy, the PCTV dose was 45 to 54 Gy. For high-grade gliomas, the prescription dose was PGTV 60 Gy and PCTV 54 Gy.

### Chemotherapy

Concurrent chemotherapy was administered with temozolomide: temozolomide 75 mg/m2, daily during radiotherapy.

Adjuvant chemotherapy: temozolomide 150–200 mg/ m2/d1-d5, every 28 days a cycle, total 6 cycles.

### Statistical analysis

The clinical characteristics of enrolled patients were used χ2 test with SPSS v22.0 (IBM, Amonk, NY, USA). The Kaplan-Meier estimator analyses were calculated the Overall Survival (ΟS) and progression-free survival (PFS) (Function Surv, R package survival, v4.2.1 (R institute for Statistical Computing, Vienna, Austria (http://www.r-project.org/))). The differences between curves were assessed using the log-rank test (function survdiff, R package survival, v4.2.1). OS was calculated from the time of histological confirmation to the date of death or loss/last time to follow-up. PFS was defined as the time interval between initiation of radiation therapy and the date of the CT or ΜRI examination that confirmed progression according to the RANO criteria [23] or related neurological symptoms or loss/last time to follow-up. The hazard ratio (HR) and the corresponding 95% confidence interval (CI) on univariate and multivariate analyses were calculated by Cox regression model. Cox proportional hazards regression was used to identify independent risk factors for OS and PFS. The 1-year OS and PFS in Subgroups were calculated using log-rank test. *P* < 0.05 (2-sided) was considered to indicate statistical significance.

### Follow-up

In the first two years, follow-up and MRI were performed every 3 months after RT, and thereafter they were performed every 6 months until tumor progression or death.

### Data availability statement

All data were presented in the manuscript and supplementary materials.

## RESULTS

### Patients characteristics

There were 67 patients diagnosed with glioma who treated in our hospital between January 2017 to December 2020. Only 45 patients met the inclusion criteria. The median follow-up time was 22 months (range, 2–108.5 months). The median age was 46 (range, 25 to 70). Of the 45 samples, 22 (48.9%) of the patients with IDH mutation, including 7 patients with astrocytoma (WHO grade 2), 6 with anaplastic astrocytoma (WHO grade 3), 2 with anaplastic oligodendroglioma (WHO grade 3) and 7 with glioblastoma (WHO grade 4). And 23 of 46 (51.1%) patients were ΙDH wild-type, including 4 astrocytoma patients (WHO grade 2), 6 anaplastic astrocytoma patients (WHO grade 3) and 13 glioblastoma patients (WHO grade 4). In IDH-mut group, there were 14 patients had MGMT methylation, 8 were MGMT-nonmethylation. For IDH-wild group, 8 of the 23 patients had MGMT methylation, while 15 patients were MGMT-nonmethylation. All patients underwent postoperative IMRT. A total of 77.3% of the patients (17 of 22) were treated with radiotherapy concurrent temozolomide in IDH-mut group, and 36.4% of the patients (8 of 22) received RT followed by chemotherapy using temozolomide. Meanwhile, all the patients received temozolomide as the concurrent chemotherapy regimen in IDH-wild group, and 11of 23 (47.8%) patients received adjuvant chemotherapy by using temozolomide after RT. The baseline characteristics of patients, stratified by IDH status, were shown in Table 1.

### Survival

**Figure 2:**
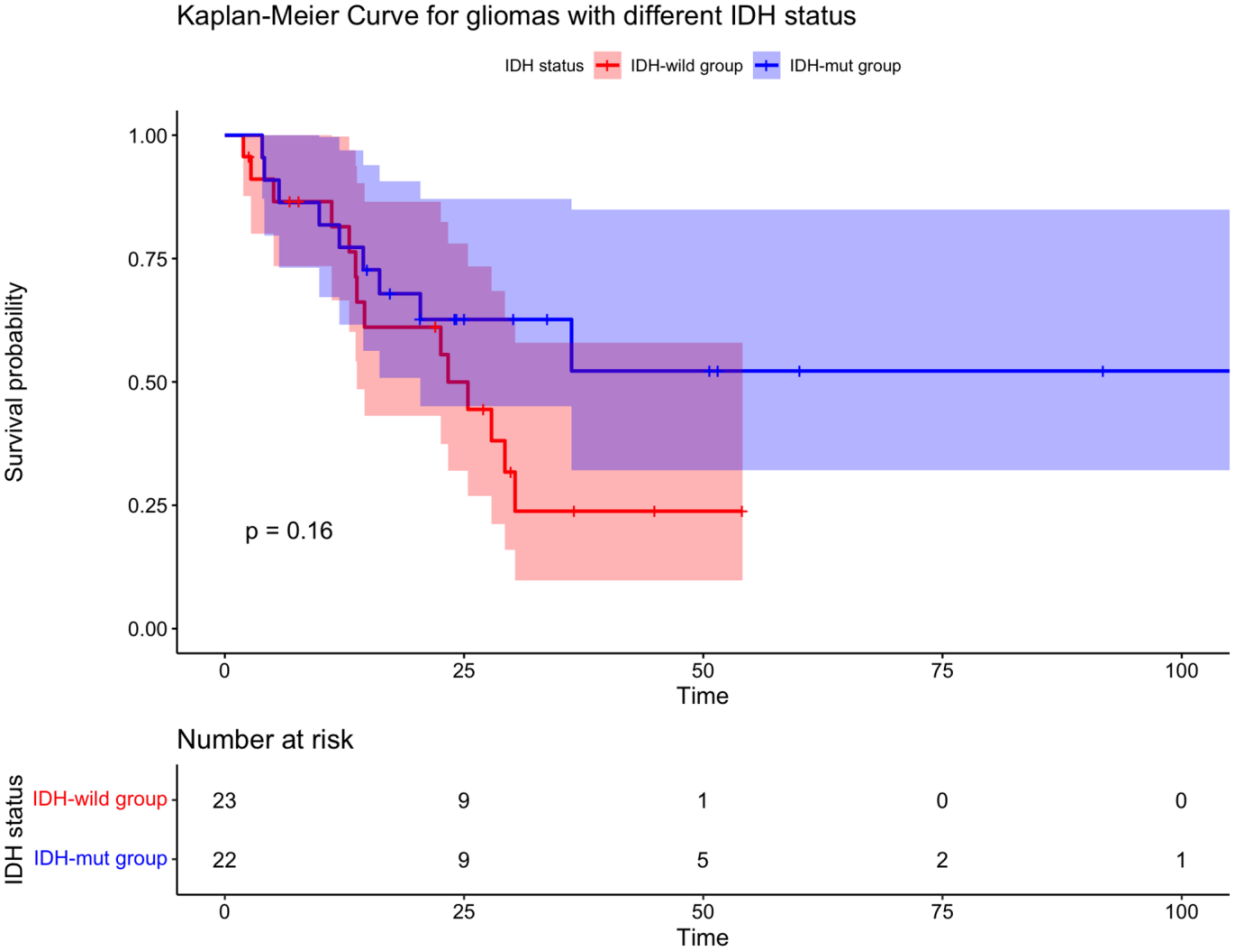
**Kaplan-Meier curve for overall survival for gliomas with different IDH status.** There is no statistically significant difference in IDH-mut group and IDH-wild group.

**Figure 3:**
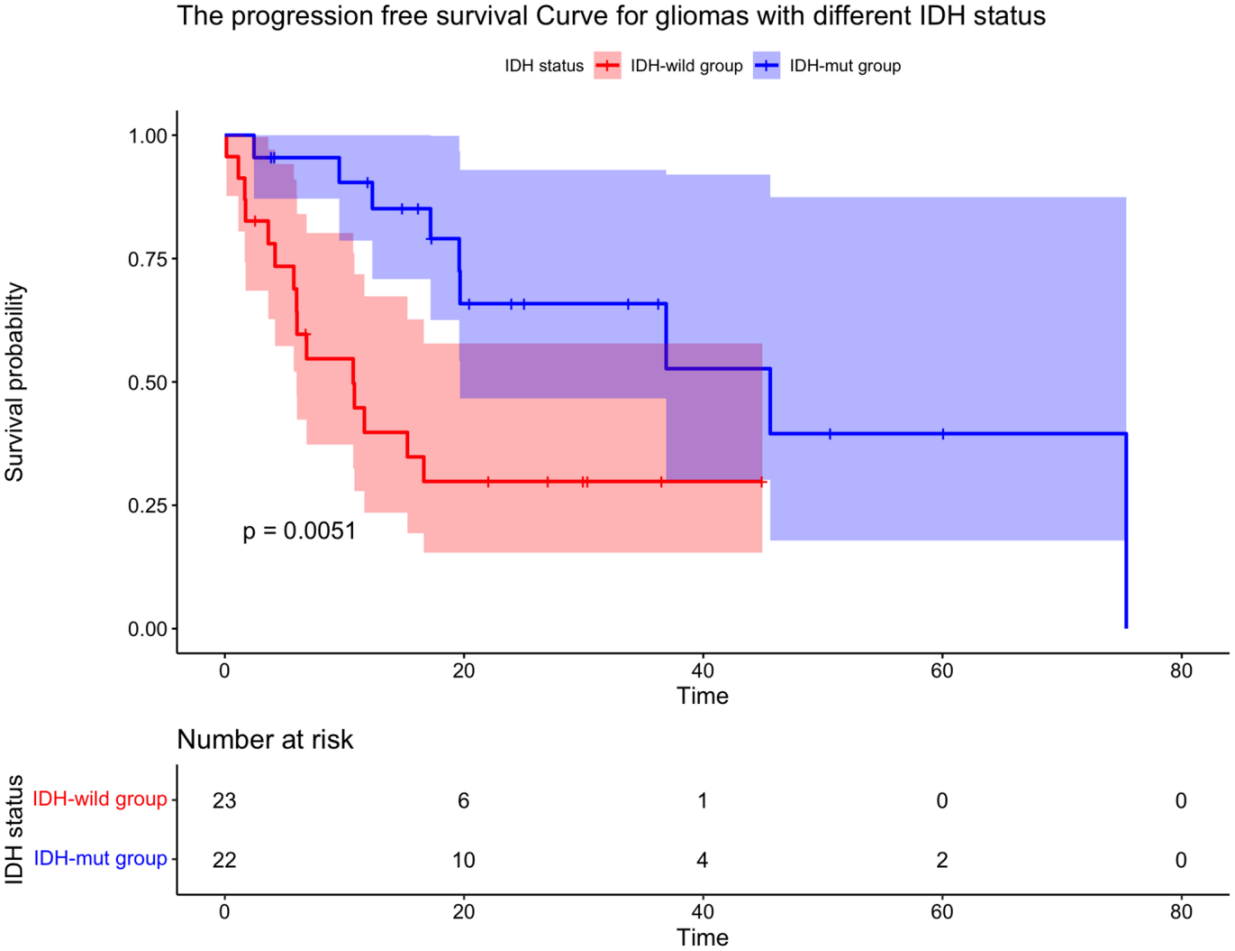
**Kaplan-Meier curve for time to tumor progression for gliomas with different IDH status.** The PFS of patients with IDH mutation was longer than that of IDH-wild type patients.

There were 24 deaths (10 of 22 (45.5%) in IDH-mut group and 14 of 23 (60.9%) in the ΙDH-wild group) and 24 patients’ recurrences (9 of 22 (40.9%) in IDH-mut group and 15 of 23 (65.2%) in the ΙDH-wild group). All patients were recurrent in radiation field. The rates of 1-year OS (81.5% vs. 77.3%, *p* = 0.16) were similar in IDH-mut group and ΙDH-wild group. While the rates of 1-year PFS (90.4% vs. 39.8%, *p* = 0.0051) were significantly higher in IDH-mut group than the ΙDH-wild group (Table 2, Figures 2, 3).

**Table 2:** The prognosis for glioma patients stratified by IDH status.

**Treatment outcomes**	**IDH-mut group (*n* = 22)**	**IDH-wild group (*n* = 23)**	**HRs (95% CI)**	***P*-value**
Locoregional failures	9 (37.5%)	15 (62.5%)		
1-year PFS	90.4%	39.8%	19.67 (0.48–38.86)	0.0051
Death	10 (41.7%)	14 (58.3%)		
1-year OS	81.5%	77.3%	4.19 (21.06–37.47)	0.16

### Survival in molecular subgroups

**Figure 4:**
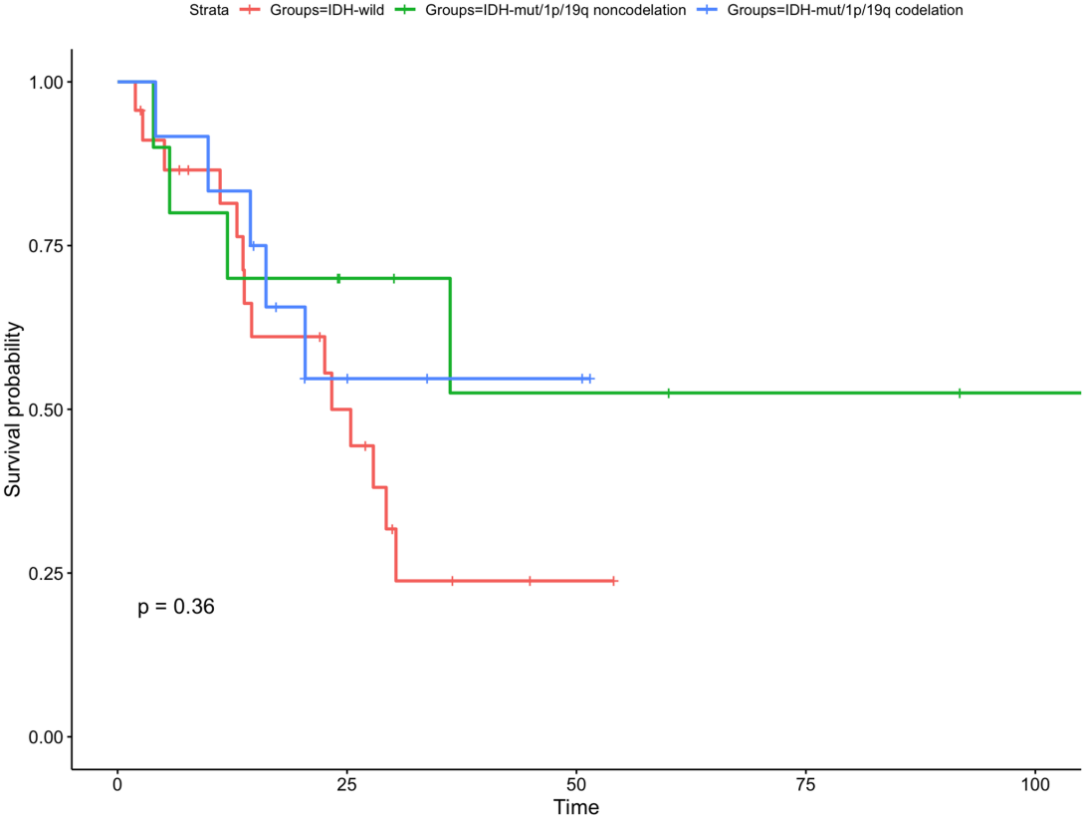
**Kaplan-Meier curve for overall survival for subgroup gliomas with different IDH status and 1p/19q codeletion status.** There is no statistically significant difference in any subgroups.

**Figure 5:**
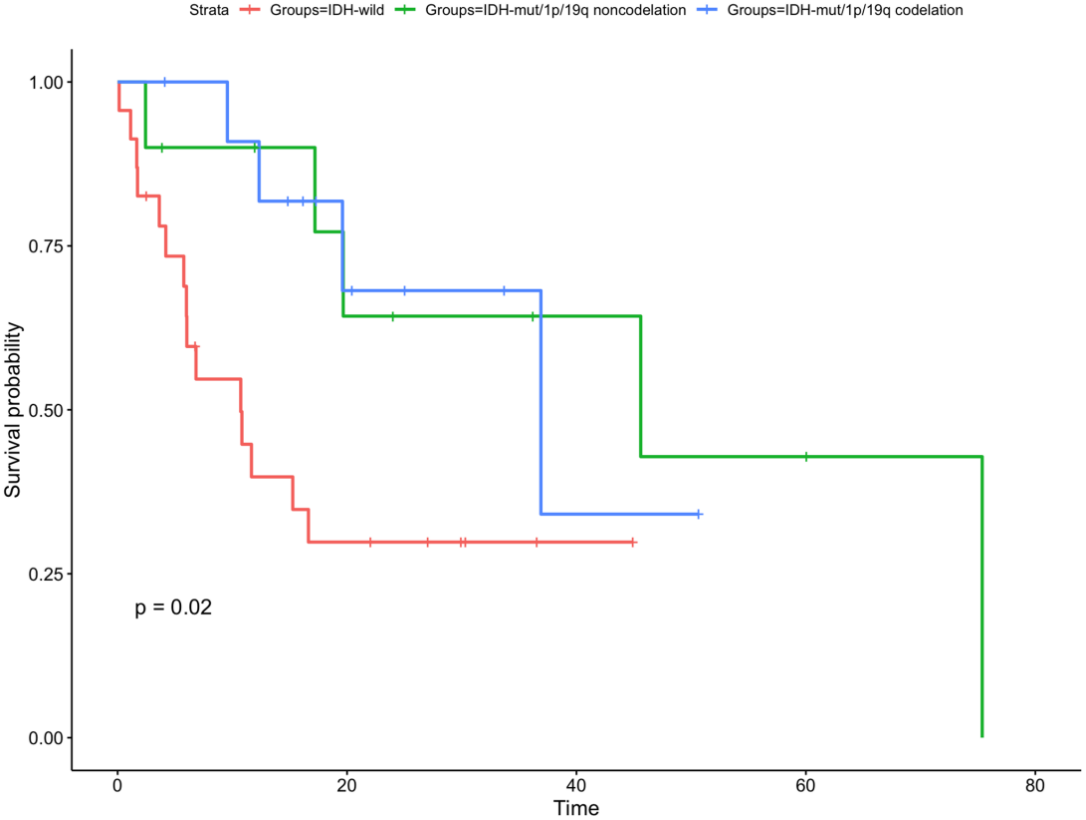
**Kaplan-Meier curve for time to tumor progression for gliomas with different IDH status.** The PFS of patients with IDH mutated/1p/19q codeletion or 1p/19q non codeletion were longer than that of IDH-wild type patients.

Of the 45 enrolled patients, there were 12 (26.7%) had IDH-mut/1p/19q codeletion, 10 (22.2%) had IDH-mut/1p/19q noncodeletion and 23 (51.1%) were IDH-wild type. The 1-year OS rate was 83.3% for IDH-mut/1p/19q codeletion group, 70.0% for IDH-mut/1p/19q noncodeletion group, and 81.5% for IDH-wild type group (*P* = 0.36, Figure 4). The rates of 1-year PFS was significantly higher in the IDH-mut/1p/19q codeletion group and IDH-mut/1p/19q noncodeletion group than in IDH-wild group (90.9% vs. 90.0% vs. 39.8%, respectively, *P* = 0.02, Figure 5).

**Figure 6:**
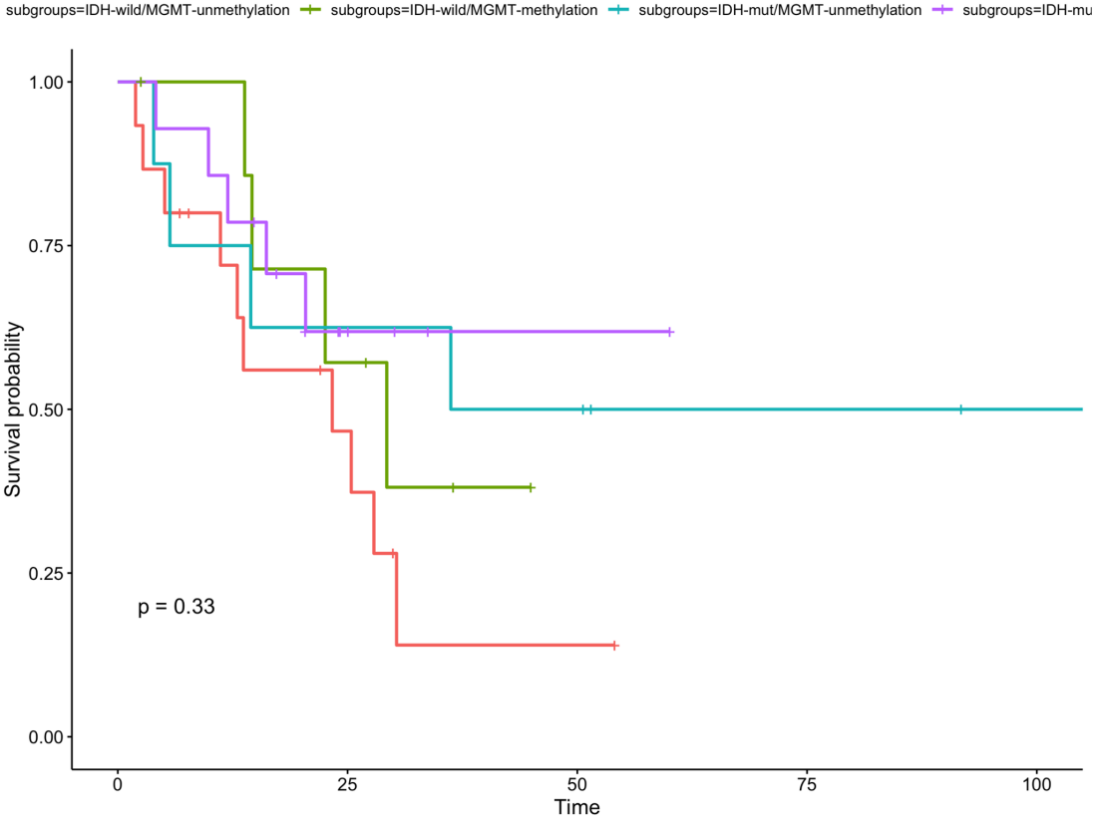
**Kaplan-Meier curve for overall survival for subgroup gliomas with different IDH status and MGMT status.** There is no statistically significant difference in the four subgroups.

**Figure 7:**
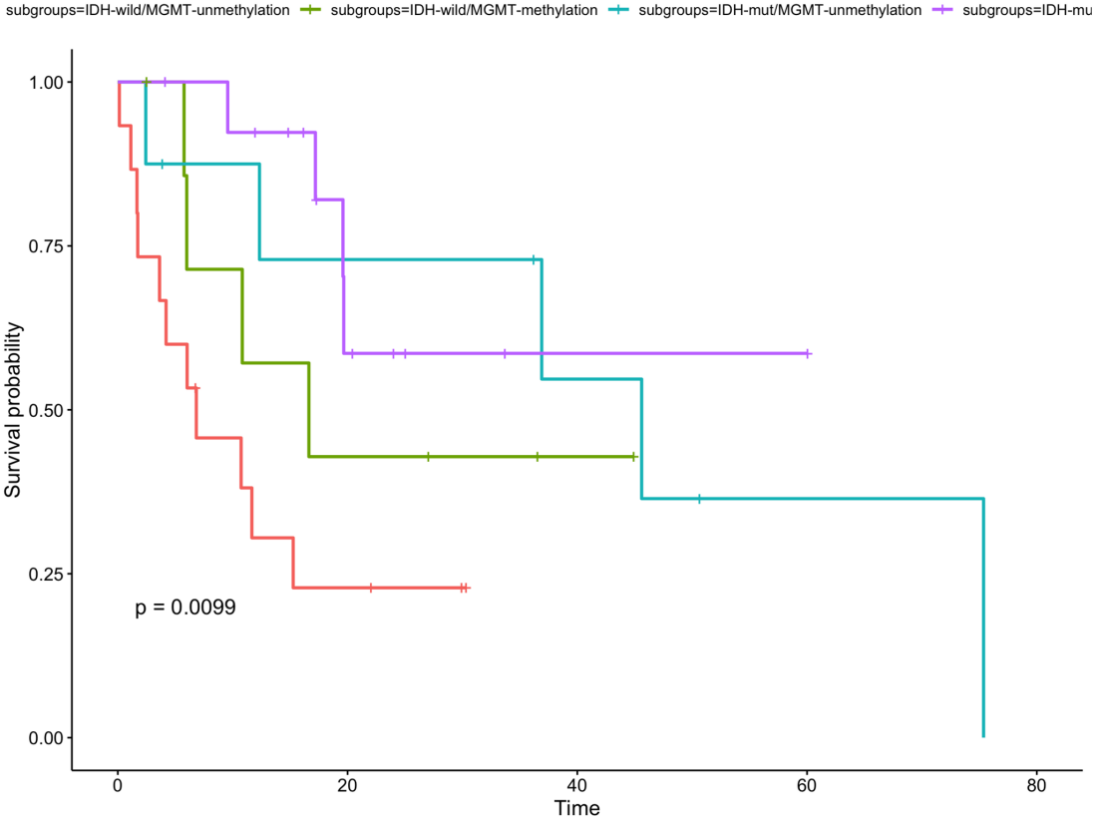
**Kaplan-Meier curve for time to tumor progression for gliomas with different IDH status and MGMT status.** The PFS of patients with IDH-mut/MGMT-methylation was the longest in the four subgroups.

Of the eligible patients profiled for the IDH status and MGMT methylation status according to the 2016 WHO classification, there were 15 (33.3%) had IDH-wild/MGMT-unmethylation, 8 (17.8%) were IDH-wild/MGMT-methylation, 8 (17.8%) were IDH-mut/MGMT-unmethylation and 14 (31.1%) were IDH-mut/MGMT-methylation. The 1-year OS rate was 72.0% for IDH-wild/MGMT-unmethylation group, 71.4% for IDH-wild/MGMT-methylation group, 75.0% for IDH-mut/MGMT-unmethylation group and 78.6% for IDH-mut/MGMT-methylation. The 4 molecular subgroups were no significantly associated with OS (*P* = 0.33, Figure 6). The 1-year PFS rate was 30.5% for IDH-wild/MGMT-unmethylation group, 57.1% for IDH-wild/MGMT-methylation group, 87.5% for IDH-mut/MGMT-unmethylation group and 92.3% for IDH-mut/MGMT-methylation. The PFS of IDH-mut/MGMT-methylation group were significantly higher than other three molecular subgroups (*P* = 0.0099, Figure 7).

Multivariate analysis of 45 valid cases showed no significant prognostic factor in OS and PFS. Univariate analysis revealed that only IDH status was significant prognostic factor of PFS (Table 3).

**Table 3:** Univariate and multivariate analyses for PFS and OS based on the clinical and molecular characteristics.

**Variable**	**No.**	**Univariate analyses**						**Multivariate analyses**					
		**PFS**			**OS**			**PFS**			**OS**		
		**HR**	**95% CI**	***P* value**	**HR**	**95% CI**	***P* value**	**HR**	**95% CI**	***P* value**	**HR**	**95% CI**	***P* value**
Gender													
Female vs. Male	15/30	0.69	0.30–1.60	0.40	0.82	0.35–1.90	0.60	0.65	0.23–1.85	0.42	0.88	0.25–3.09	0.84
Age													
≤40 vs. >40	16/29	1.76	0.70–4.39	0.22	2.31	0.93–5.75	0.07	2.87	0.56–14.84	0.19	2.61	0.45–15.04	0.27
WHO													
IV vs. III	20/14	1.00	0.41–2.47	0.20	0.78	0.31–1.95	0.20	2.11	0.54–8.18	0.20	0.89	0.15–5.20	0.20
IV vs. II	20/11	0.34	0.09–1.19		0.35	0.10–1.24		0.70	0.09–5.12		0.16	0.01–3.15	
ECOG													
1 vs. 0	41/4	0.72	0.17–3.09	0.66	0.86	0.20–3.69	0.80	1.80	0.15–21.49	0.64	6.82	0.18–257.33	0.27
Tumor site													
Cerebellum vs. Temporal lobe	1/18/	19416186		0.43	0.24	0.02–2.06	0.10	2.378e+07	0.00–Inf	0.33	0.04	0.00–2.46	0.10
Cerebellum vs. Occipital lobe	1/2	118719136			1.05	0.06–17.19		1.835e+08	0.00–Inf		0.09	0.00–15.51	
Cerebellum vs. Parietal lobe	1/8	15570644			0.23	0.02–2.10		2.827e+07	0.00–Inf		0.05	0.00–4.77	
Cerebellum vs. Frontal lobe	1/16	12113679			0.12	0.01–1.11		1.029e+07	0.00–Inf		0.01	0.00–0.70	
Pathology													
Glioblastoma vs. anaplastic oligodendroglioma	20/2	0.97	0.12–7.54	0.26	2.67	0.58–12.25	0.09	6.48	0.24–177.87	0.28	36.85	0.48–2828.25	0.08
Glioblastoma vs. anaplastic astrocytomas	20/12	1.01	0.39–2.58		0.60	0.21–1.69		NA	NA		NA	NA	
Glioblastoma vs. astrocytoma	20/11	0.34	0.09–1.19		0.35	0.10–1.23		NA	NA		NA	NA	
Tumor size													
≥6 vs. <6	19/26	0.45	0.19–1.09	0.07	0.50	0.22–1.15	0.10	0.67	0.20–2.25	0.52	0.49	0.11–2.21	0.35
Resection													
Biospy vs. total resection	2/13	13641627		0.07	0.59	0.08–4.50	0.60	1.007e+07	0.00–Inf	0.61	0.06	0.00–1.79	0.30
Biospy vs. subtotal resection	2/30	35172362			0.37	0.04–3.28		1.734e+07	0.00–Inf		0.07	0.00–1.94	
Chemotherapy													
No vs. Yes	5/40	1.71	0.40–7.31	0.47	0.97	0.29–3.26	1.00	0.64	0.03–12.58	0.77	0.24	0.01–9.04	0.42
Adjuvant chemotherapy													
No vs. Yes	26/19	1.29	0.56–2.96	0.55	0.85	0.37–1.98	0.70	1.19	0.32–4.38	0.79	0.56	0.12–2.72	0.48
IDH status													
Wild vs. IDH mutation	23/22	0.29	0.12–0.73	0.005	0.56	0.24–1.29	0.20	0.42	0.06–3.18	0.39	0.36	0.02–5.57	0.44
1p/19q codelation													
No vs. Yes	32/13	0.38	0.13–1.12	0.07	0.68	0.25–1.86	0.50	0.28	0.03–2.43	0.24	0.16	0.01–1.89	0.11
MGMT methylation													
No vs. Yes	23/22	0.45	0.19–1.07	0.06	0.64	0.27–1.49	0.30	0.36	0.09–1.43	0.13	0.35	0.05–2.54	0.27

## DISCUSSION

The WHO classification of brain tumor in version 1,2016 [4] proposed molecular characteristics as one of the most important characteristics for gliomas, especially isocitrate dehydrogenase (IDH) 1/2, which is strongly associated with the prognosis and related to tumor grade. Molecular features become more important to gliomas. In the cIMPACT-NOW Consortiun for taxonomy of primary brain tumors suggested to reclassify those patients who had IDH wild-type diffuse gliomas as diffuse astrocytic gliomas, IDH wild-type with molecular features of glioblastoma, WHO 4 [24, 25]. Some of the researches also confirmed that patients who had IDH wild-type display a poor survival as patients with IDH wild-type glioblastoma [26]. Most of the previous studies based on the conventional radiation technology or 3D-CRT, with the development of radiotherapy technology, are these advances bring some benefit for glioma patients based on the molecular characteristics? Our study retrospectively collected glioma patients regardless of their pathology, clinical features, surgical information and adjuvant treatments, reclassified the patients based on their molecular characteristics. In our study, patients with IDH mutated had better PFS than those in IDH-wild group ((90.4% vs. 39.8%), but did not significantly differ in OS between the two groups. These results were similar to Michael Weller et al. ’s founding [19]. In the study of Qi SongTao, he also found that IDH mutation, MGMT promoter methylation and 1p19q codeletion were related to prolong PFS [27]. However, there were some different results. Marc Sanson et al. found patients (including grade 2, 3 and 4 gliomas) with IDH-mutated had better OS and PFS than non-mutated tumors [28]. C. Houillier et al. also revealed that IDH mutation and 1p19q codeletion were associated with prolonged overall survival [29]. We summarized the reason that our results didn’t get the significantly differences in OS may because our study is a small sample size and the follow-up time is too short or may be the OS benefits from the progress of radiation technology (e.g., IMRT), but not the molecular features. In our research, we also observed that IDH-mut/1p/19q codeletion group and IDH-mut/1p/19q noncodeletion group patients had longer PFS than patients with IDH-wild type (90.9% vs. 90.0% vs. 39.8%). In the subgroups reclassified by IDH and MGMT status, the results revealed similar survival in whole groups for patients all received IMRT radiotherapy technology. Early in 2005, Monika E. Hegi et al. found that glioblastoma patients with MGMT methylation treated with temozolomide had longer OS [30]. Although our study had some flaws, but the results demonstrated the prognosis of patients with different molecular types of gliomas under the same radiation technology. It more powerful to distinguish whether the progress of technology or molecular characteristics benefit for glioma patients.

In conclusion, this retrospective study showed that patients with IDH mutation had better PFS than those patients with IDH-wild type. In terms of IDH-mut/1p/19q codeletion and IDH-mut/1p/19q noncodeletion of glioma patients, the outcomes were shown longer PFS than patients with IDH-wild type, but no difference in OS among the subgroups. Patients with IDH-mut/MGMT-methylation had the best prognosis in the subgroups. However, these results still needs further confirmation of large sample size, prospectively, randomized controlled trails.
